# Oil candidate genes in seeds of cotton (*Gossypium hirsutum* L.) and functional validation of *GhPXN1*

**DOI:** 10.1186/s13068-023-02420-1

**Published:** 2023-11-06

**Authors:** Chenxu Gao, Xiao Han, Zhenzhen Xu, Zhaoen Yang, Qingdi Yan, Yihao Zhang, Jikun Song, Hang Yu, Renju Liu, Lan Yang, Wei Hu, Jiaxiang Yang, Man Wu, Jisheng Liu, Zongming Xie, Jiwen Yu, Zhibin Zhang

**Affiliations:** 1 Zhengzhou Research Base, National Key Laboratory of Cotton Bio-Breeding and Integrated Utilization, Zhengzhou University, Zhengzhou, 450000 China; 2 National Key Laboratory of Cotton Bio-Breeding and Integrated Utilization, Institute of Cotton Research, Chinese Academy of Agricultural Sciences, Anyang, 455000 China; 3 Key Laboratory of Cotton Biology and Genetic Breeding in the Northwest Inland Cotton Production Region of the Ministry of Agriculture and Rural Affairs, Institute of Cotton Research, Xinjiang Academy of Agricultural and Reclamation Science, Shihezi, 832000 China; 4 Shijiazhuang Academy of Agriculture and Forestry Sciences, Shijiazhuang, 050000 China; 5 Jiangsu Academy of Agricultural Sciences, Nanjing, 210000 China

**Keywords:** *Gossypium hirsutum* L., Cottonseed oil content, Comparative transcriptome, Fatty acid biosynthesis, Gene introgression

## Abstract

**Background:**

Cottonseed oil is a promising edible plant oil with abundant unsaturated fatty acids. However, few studies have been conducted to explore the characteristics of cottonseed oil. The molecular mechanism of cottonseed oil accumulation remains unclear.

**Results:**

In the present study, we conducted comparative transcriptome and weighted gene co-expression network (WGCNA) analysis for two *G. hirsutum* materials with significant difference in cottonseed oil content. Results showed that, between the high oil genotype 6053 (H6053) and the low oil genotype 2052 (L2052), a total of 412, 507, 1,121, 1,953, and 2,019 differentially expressed genes (DEGs) were detected at 10, 15, 20, 25, and 30 DPA, respectively. Remarkably, a large number of the down-regulated DEGs were enriched in the phenylalanine metabolic processes. Investigation into the dynamic changes of expression profiling of genes associated with both phenylalanine metabolism and oil biosynthesis has shed light on a significant competitive relationship in substrate allocation during cottonseed development. Additionally, the WGCNA analysis of all DEGs identified eight distinct modules, one of which includes *GhPXN1*, a gene closely associated with oil accumulation. Through phylogenetic analysis, we hypothesized that *GhPXN1* in *G. hirsutum* might have been introgressed from *G. arboreum*. Overexpression of the *GhPXN1* gene in tobacco leaf suggested a significant reduction in oil content compared to the empty-vector transformants. Furthermore, ten other crucial oil candidate genes identified in this study were also validated using quantitative real-time PCR (qRT-PCR).

**Conclusions:**

Overall, this study enhances our comprehension of the molecular mechanisms underlying cottonseed oil accumulation.

**Supplementary Information:**

The online version contains supplementary material available at 10.1186/s13068-023-02420-1.

## Background

Edible oil and biodiesel are becoming increasingly scarce with the population growth and deterioration of climatic conditions in the world [1, 2]. Cotton (*Gossypium hirsutum* L.), as the fourth-largest oil crop, is not only an excellent source of renewable fiber, but also an important source of vegetable oil and biodiesel [3]. Cottonseed oil is an important by-product of cotton, and contains a large number of unsaturated fatty acids (more than 50% linoleic acid), which is beneficial to human health [4]. As the neutral flavor that will not mask food flavors, cottonseed oil is ideal for frying and fine cooking in the food industry [5]. Moreover, suitable carbon chain length in cottonseed oil is considered as an ideal biofuel feedstock [6, 7]. The biosynthesis and accumulation of cottonseed oil mainly involve four stages, namely the conversion of sucrose to pyruvate, de novo fatty acid (FA) synthesis, endoplasmic triacylglycerol (TAG) synthesis, and oil-body assembly [8]. Sucrose is the primary carbon source for seed storage oil biosynthesis [9]. De novo FA synthesis is catalyzed by a complex of several enzymes in plastids [10]. Briefly, free FA chains are released from FA acyl carrier protein (acyl-ACP) under the catalysis of FA acyl-ACP thioesterase in plastids, and then transported into the endoplasmic reticulum (ER) for functional modifications, such as the elongation and desaturation of acyl chains [11]. The modified FA chain is further processed into TAGs by the Kennedy pathway [12–14]. Another TAG synthesis pathway is an acyl-CoA-independent pathway mediated by phospholipids:diacylglycerols acyltransferase (PDAT) transfer fatty acyl moieties from phospholipids (PL) to diacylglycerol (DAG), then to form TAGs [15].

The phenylpropanoid metabolism process generally synthesis many secondary metabolites, including lignins and flavonoids, based on the few intermediates of the shikimate pathway. Phenylpropanoid homeostasis is achieved by modulating metabolic flux redirections linked to oil synthesis [16]. With the development of next-generation sequencing (NGS) technologies, an increasing number of studies on oil traits have been conducted in various crops. For instance, a novel key regulatory gene *GhCYSD1* is associated with oil synthesis [10]. Integration of comparative transcriptome and population mapping identified 21 candidate genes associated with oil accumulation in *G. barbadense* and *G. hirsutum*, such as *GbSWEET* and *GbACBP6* [6], *GhKASII* [17], *GhSAD* [18], *GhWRI1* [19] and *GmPEPC1* [20]. In addition, comparative transcriptome analysis during seed development stages of soybean [21–23], sesame [24], peanut [25, 26] and *Brassica napus* [27–29] have also been studied. Although some studies about oil content have been reported, further studies are still needed compared to other agronomic traits.

In order to explore the molecular mechanisms of oil synthesis and accumulation during cottonseed development, comparative transcriptome analysis and WGCNA analysis were performed between two cotton varieties with significant difference in seed oil content in this study. Results showed that the phenylpropanoid biosynthesis is antagonistic to oil biosynthesis. Moreover, *GhPXN1*, an introgression gene from *G. arboreum* into *G. hirsutum*, were found to be associated with cottonseed oil content, which was also confirmed by overexpression in tobacco leaf. Understanding the genetic mechanisms underlying cottonseed oil accumulation can contribute to breeding improvement of high oil cotton varieties.

## Results

### Seed oil content of *G. hirsutum* H6053 and L2052

Based on the *G. hirsutum* RIL population in our laboratory, it was observed that the Best Linear Unbiased Prediction (BLUP) value for cottonseed oil content differed significantly between *G. hirsutum* H6053 (34.97%) and L2052 (26.53%) across ten distinct environmental conditions. Furthermore, there were significant variations in the proportions of FA components between H6053 and L2052. Hence, the two genotypes, namely H6053 and L2052, were selected for the comparative transcriptional analysis (Additional file 1: Table S1).

### Transcriptome analysis of *G. hirsutum* H6053 and L2052

Transcriptomic analysis of H6053 and L2052 was performed using samples collected from five different stages of cottonseed development, including 10, 15, 20, 25, and 30 day post anthesis (DPA). Principal component analysis (PCA) of all samples was carried out based on gene expression level at different stages, and the result showed that samples from the same stage consistently clustered together (Additional file 2: Fig. S1a), indicating that the RNA-seq data were suitable for the intended comparative transcriptome analysis.

### Identification and annotation of DEGs

**Fig. 1:**
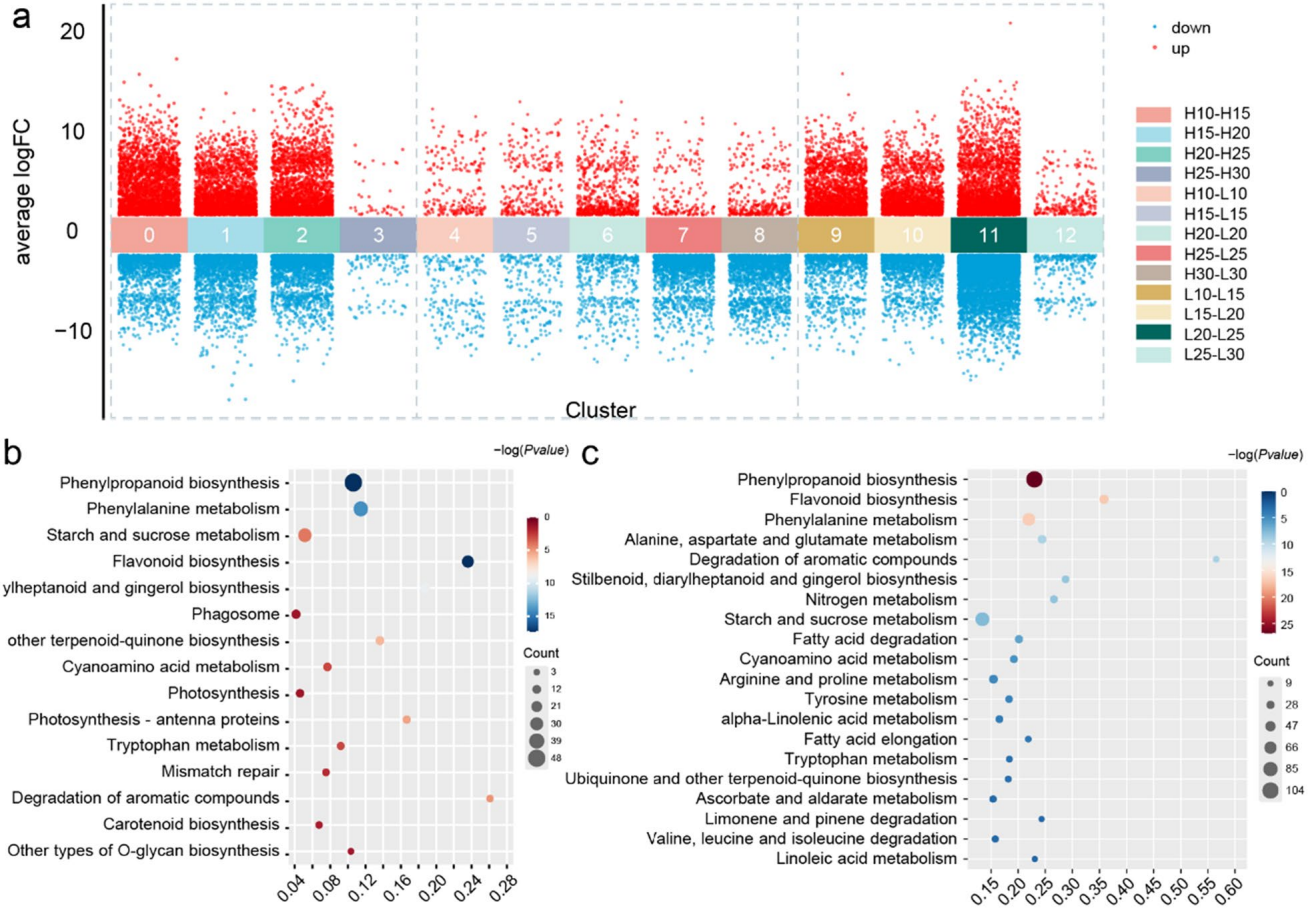
**a** Comparative transcriptomic analysis between H6053 and L2052 at different stages (10, 15, 20, 25, 30 DPA) of cottonseed development. Red scatter plot indicates up-regulation DEGs; blue scatter plot indicates down-regulation DEGs. There is total 13 clusters, from cluster 0 to cluster 12. **b**, **c** KEGG pathway enrichment analysis of '25DPA vs 20DPA' down-regulated DEGs in cluster 2 (H20-H25) and cluster 11 (L20-L25), respectively

Time-series differential expression analysis of genes was conducted to gain a comprehensive understanding of their impact on cottonseed oil accumulation. Notably, a total of 414, 507, 1,121, 1,953, and 2,019 DEGs were discovered at different stages (10, 15, 20, 25, and 30 DPA) between H6053 and L2052, respectively (Fig. 1a, Additional file 2: Fig. S1b), with a noticeable rapid increase in DEGs observed from 20 DPA onwards. Among the up-regulated genes, a significant proportion was found at 20 DPA, particularly within Cluster 11 (668 genes) (Fig. 1a), with 517 genes specifically up-regulated in H6053 compared to L2052 (Cluster 2). KEGG enrichment analysis of these up-regulated genes revealed significant enrichment in pathways such as limonene and pinene degradation, cutin, suberin, and wax biosynthesis (Additional file 2: Fig. S1c). Conversely, the down-regulated genes were predominantly found at 25 DPA and 30 DPA, and KEGG enrichment analysis indicated they were mainly enriched in the phenylpropanoid biosynthesis and phenylalanine metabolism pathways (Additional file 2: Fig. S1d). Here, we mainly focused on the down-regulated DEGs. Comparing the down-regulated DEGs at the same stage between H6053 and L2052, the most substantial difference was observed between Cluster 2 (H20-H25) and Cluster 11 (L20-L25), with 2,200 down-regulated genes in L2052 and 5440 down-regulated genes in H6053 (Fig. 1a). KEGG pathway enrichment analysis of these down-regulated DEGs in cluster 2 and cluster 11 were carried out and results showed that 15 and 44 pathways were enriched for genes in H6053 (Fig. 1b) and L2052 (Fig. 1c), respectively (Additional file 1: Table S2), with 12 pathways being shared between the two genotypes. These pathways include carotenoid biosynthesis, flavonoid biosynthesis, flavone and flavanol biosynthesis, phenylpropanoid biosynthesis, and metabolism of phenylalanine. Notably, the most significant enrichment pathway is phenylpropanoid biosynthesis.

### Dynamic expression profiles of genes associated with phenylpropanoid synthesis

**Fig. 2:**
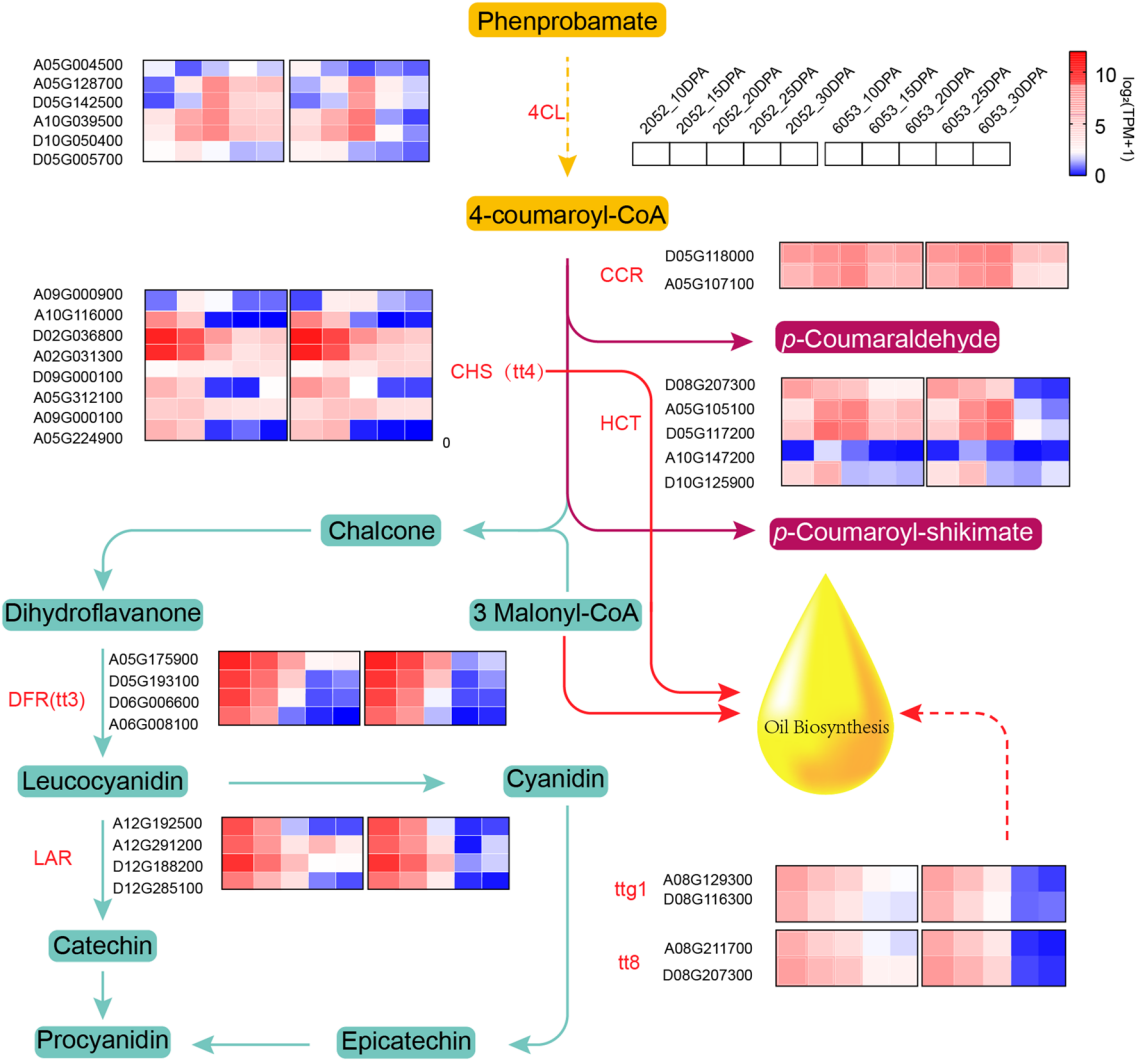
Expression profile of genes involved in the phenprobamate pathway in development cottonseed. The expression level is highlighted with gradient color in 10, 15, 20, 25, and 30 DPA. The left and right corners represent L2052 and H6053, respectively

Phenylpropanoid biosynthesis represents a crucial secondary metabolic pathway that is closely associated with seed oil content [30]. In order to explore the underlying mechanisms influencing seed oil content, we conducted a comparative analysis of gene expression profiles related to phenylpropanoid metabolism during cottonseed development in both genotypes, with a specific emphasis on flavonoid synthesis. The results revealed relatively higher expression levels of genes related to flavonoid biosynthesis in L2052 compared to H6053 throughout the flavonoid synthesis process (Fig. 2). 4-Coumarate-CoA ligase (4CL) is an enzyme responsible for the formation of 4-coumaroyl CoA, a key substrate in flavonoid synthesis [31]. Interestingly, we observed significant down-regulated expression of 4CL encoding gene in H6053 relative to L2052 during phenylpropanoid synthesis. Additionally, the genes *TTG1* and *TT8*, which synergistically regulate flavonoid biosynthesis, also displayed significant down-regulation in H6053 compared to L2052. Further analysis revealed a normal distribution of catechin content in cotton natural populations (Additional file 2: Fig. S2a), with a negative correlation observed between catechin content and both linoleic acid (Additional file 2: Fig. S2b) and total oil content (Additional file 2: Fig. S2c). Conversely, a positive correlation was observed between catechin content and C18:3 (Additional file 2: Fig. S2d). These findings shed light on the intricate relationships and potential regulatory mechanisms involved in the phenylpropanoid pathway and its impact on seed oil content.

### Dynamic expression profiles of genes involved in oil biosynthesis

**Fig. 3:**
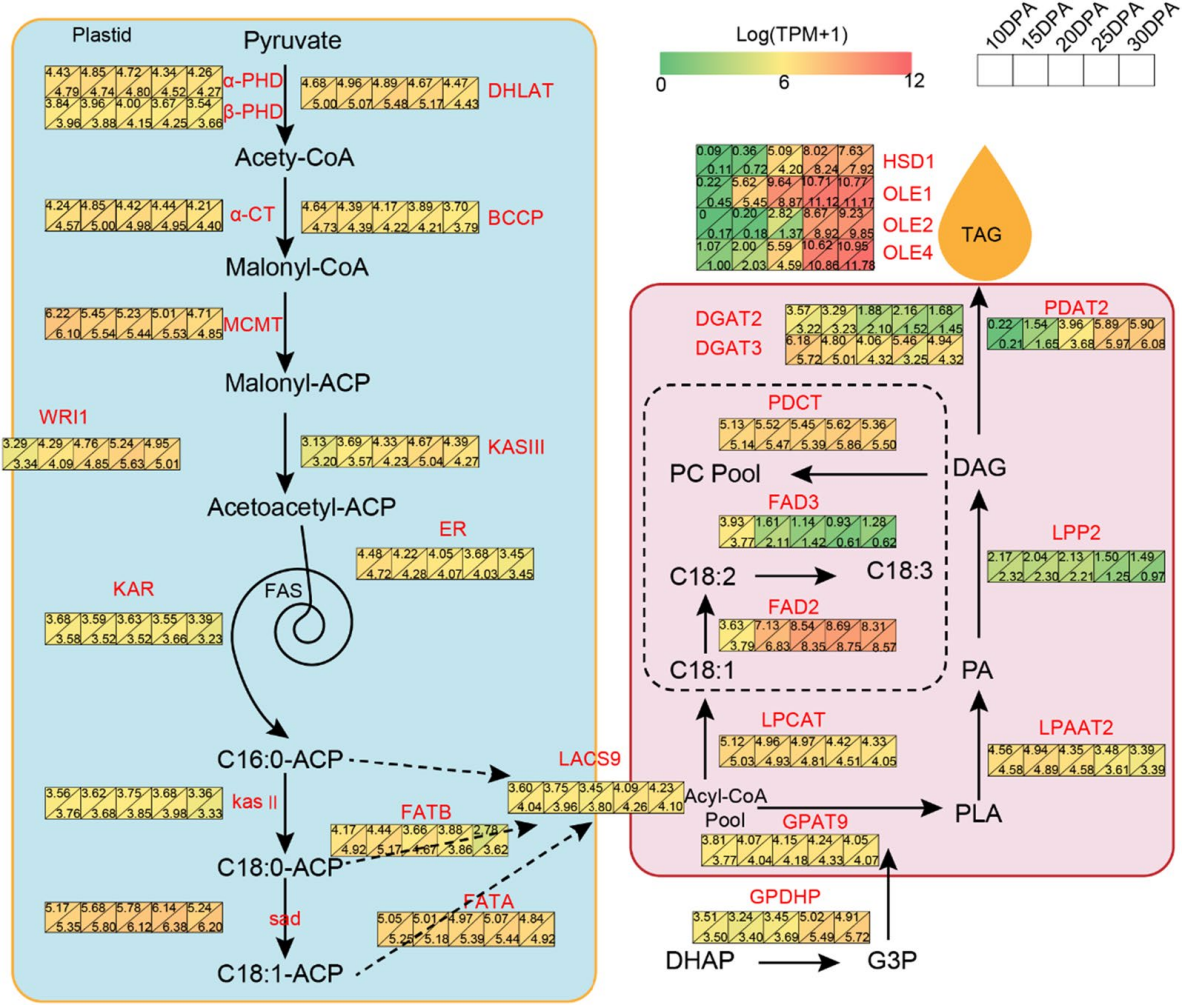
Expression profile of genes involved in cottonseed oil biosynthesis. The expression level is highlighted with gradient color in 10, 15, 20, 25, and 30 DPA. The upper left and lower right corners represent L2052 and H6053, respectively

To elucidate the potential mechanisms underlying the variation in cottonseed oil synthesis, we further investigated the expression of genes related to oil biosynthesis during cotton ovule development. Remarkably, the peak period of cottonseed oil content in H6053 occurred earlier (at 25 DPA) compared to L2052 (at 30 DPA). Based on previous reporters [6, 10], 330 genes were identified involving in oil synthesis (Additional file 1: Table S3). Notably, the expression levels of these genes were consistently higher in H6053 compared to L2052 during cotton ovule development (Fig. 3). Particularly, several genes encoding oil body proteins (OLE1, OLE2, and OLE4) exhibited elevated expression levels during the later stages of cottonseed development. Among the key enzymes responsible for TAG assembly, namely GPAT, LPAAT, LPACT, and PDAT [11–13], the expression level of GPAT remained relatively stable across different time periods. Interestingly, the expression level of the PDAT gene increased gradually with cotton ovule development, while the expression trends of LPAAT and LPCAT were opposite to each other.

Transcription factors (TFs) play vital roles in the regulation of gene expression and are pivotal in plant growth and development. In this study, a total of 59 genes belonging to 14 TFs families were identified. Intriguingly, only 20% of these TFs exhibited significant expression (TPM > 10) at 10 DPA, while 75% of TFs expressed at 20 DPA (Additional file 2: Fig. S3a, Additional file 1: Table S4). Notably, TFs WRI1 and NF-YB6 exhibited high expression levels during the rapid oil accumulation stage (20 DPA to 30 DPA) in H6053 (Additional file 2: Fig. S3b).

### Gene co-expression network analysis with WGCNA

**Fig. 4:**
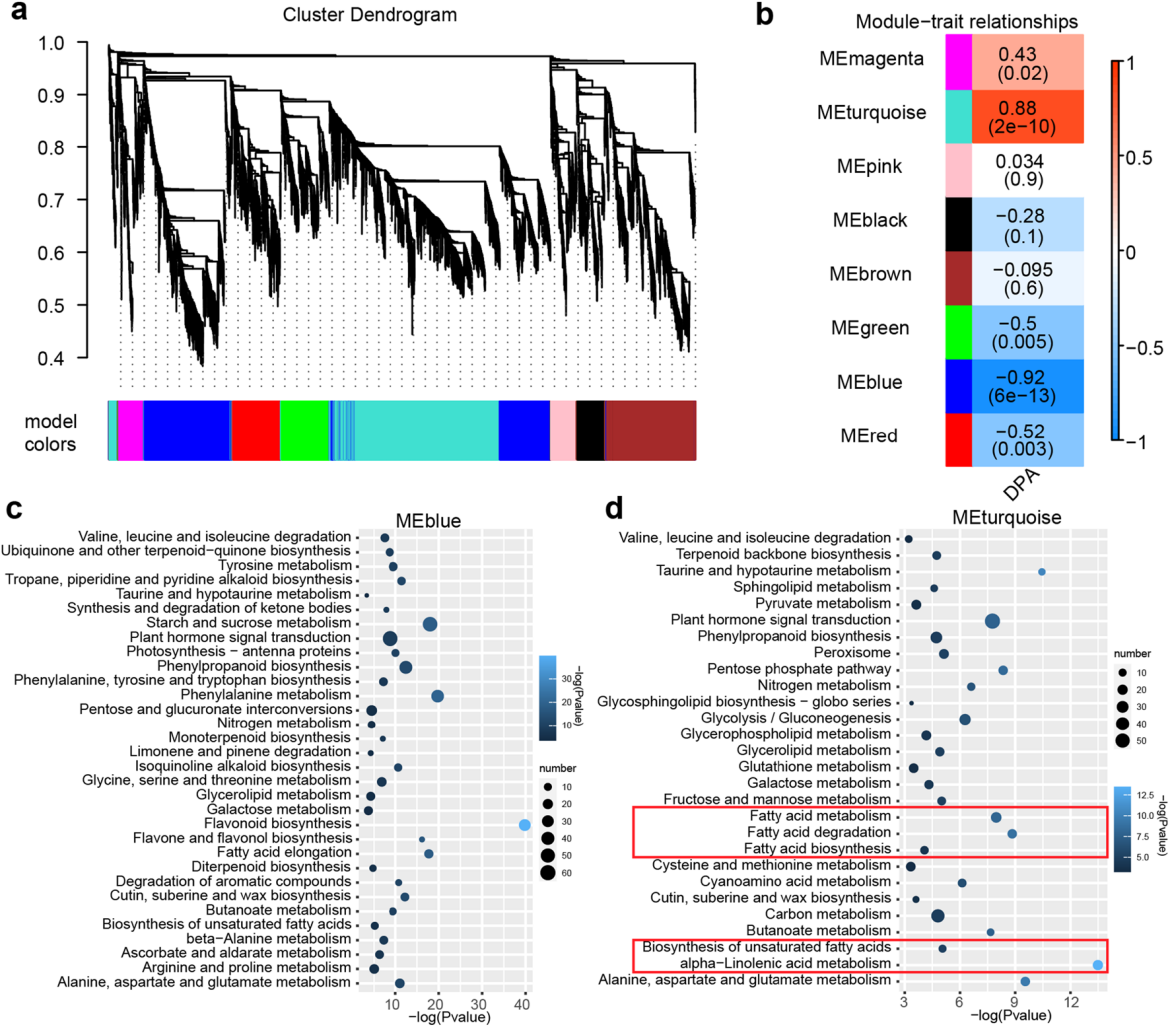
Network analysis dendrogram showing co-expression modules identified by WGCNA of DEGs. **a** Dendrogram plot with color annotation. The genes in the same branch could be assigned to different modules. The main tree branches form eight modules labeled with different colors. **b** Module-sample association. Each row corresponds to a module. Each column corresponds to a specific material. The color of each cell at the row-column intersection indicates the coefficient of correlation between the module and the material. A high degree of correlation between a particular module and the material is indicated by the red color. **c**, **d** Significantly enriched KEGG signaling pathways of differentially expressed genes derived from modules MEblue and MEturquoise, respectively

A total of 10,914 DEGs were selected for WGCNA analysis with an empirical soft threshold set at eight. Consequently, these genes were categorized into eight modules specific to different cotton varieties and developmental stages (Fig. 4a, b). Here, we used cotton ovules development stages “DPA” as the phenotype for WGCNA analysis. Results showed that MEblue and MEturquoise modules were closely corrected to oil content traits. KEGG pathway enrichment analysis of genes in the two modules were performed, and results suggested that the genes in MEblue module were mainly significantly enriched in phenylpropane and flavone metabolism (Fig. 4c), while genes in MEturquoise module were mainly enriched in FA metabolism (Fig. 4d). In MEturquoise module, gene *GhPXN1* (*Gh_A10G238500*), encoding peroxisomal membrane protein, had the most significant expression difference during 20–30 DPA between H6053 and L2052. Moreover, GWAS analysis of a NAM cotton population also found that *GhPXN1* associated with cottonseed oil content (unpublished data). KEGG pathway enrichment analysis of genes in the other four modules was also conducted (Additional file 2: Fig. S4).

### Overexpression of *GhPXN1* reduced the oil content

**Fig. 5:**
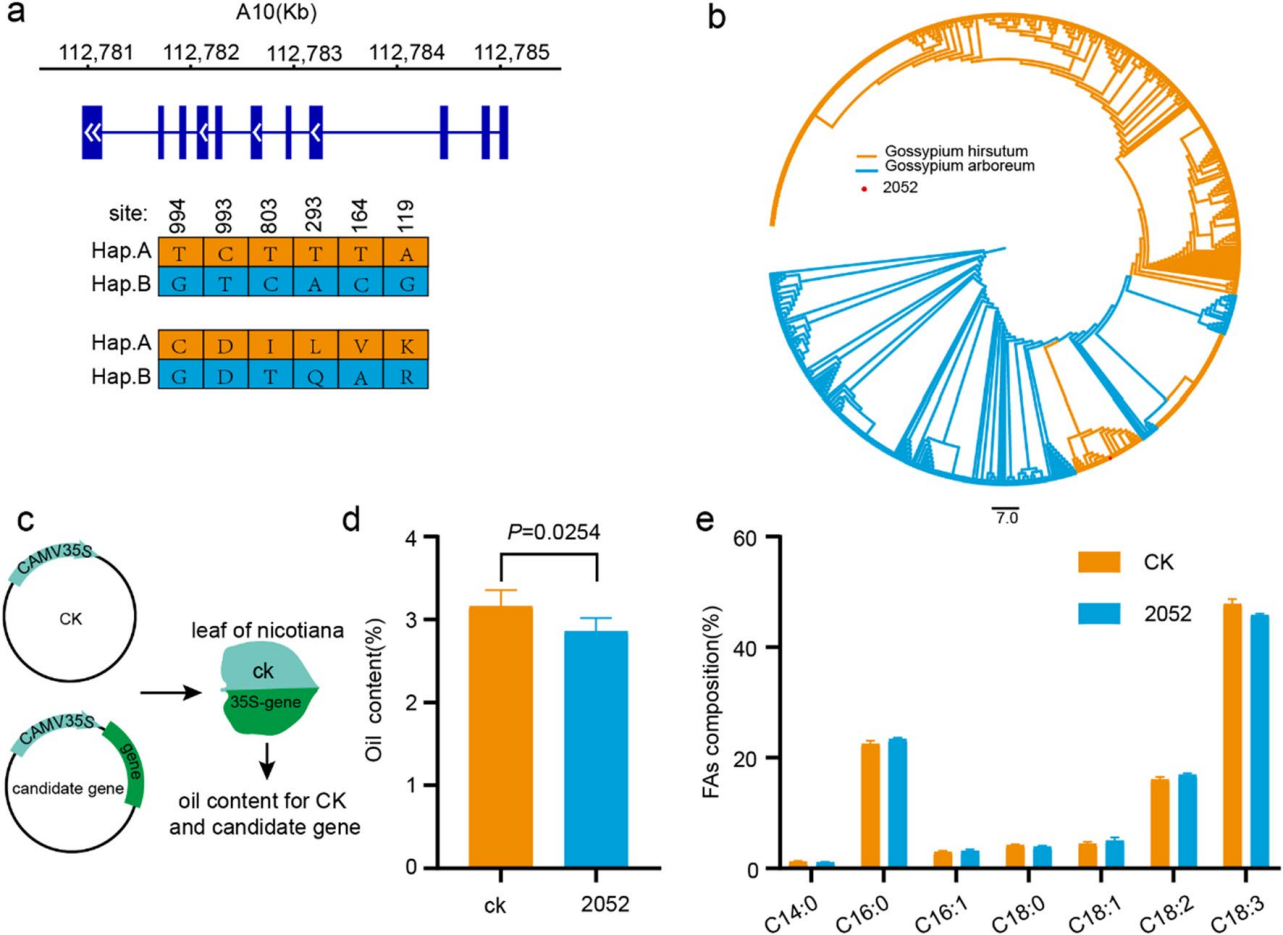
**a** Sequence amplification diagram of the *GhPXN1* gene clone. Hap.A and Hap.B represent H6053 and L2052, respectively. **b** Phylogenetic tree of *GhPXN1* genes from 208 *G. arboreum* accessions and 380 *G. hirsutum* accessions (including L2052). **c** Overexpression of the *GhPXN1* gene in tobacco. The comparison of the total oil content (**d**) and FA composition (**e**) in the leaves between the control (CK) and the leaves with 35S-gene

The *GhPXN1* gene was successfully cloned and its sequence was determined. Sequence alignment analysis identified six single nucleotide polymorphisms (SNPs) between L2052 and H6053, which resulted in five amino acid changes in the *GhPXN1*-coding protein (Fig. 5a). A phylogenetic tree was constructed using *GhPXN1* genes from 208 *G. arboreum* accessions [32], 380 *G. hirsutum* accessions [33] and *G. hirsutum* 2052. The result showed that *GhPXN1* gene in L2052 was grouped in the branch of *G. arboreum* accessions, which indicating that *G. hirsutum* gene *GhPXN1* may have introgressed from *G. arboreum* (Fig. 5b). Furthermore, ectopic overexpression of *GhPXN1* gene (2300-*GhPXN1*) was performed in tobacco leaves (Fig. 5c), leading to a significant reduction in oil content in tobacco leaves (2.86%) compared to empty-vector transformants (3.16%) (Fig. 5d). Additionally, the analysis of major FA compositions including myristic acid (C14:0), palmitic acid (C16:0), palmitoleic acid (C16:1), stearic acid (C18:0), oleic acid (C18:1), linoleic acid (C18:2), and linolenic acid (C18:3) in the leaves revealed that the overexpression of *GhPXN1* led to a decreased relative proportion of linolenic acid (C18:3) compared to the empty vector transformants (Fig. 5e).

### qRT-PCR validation

**Fig. 6:**
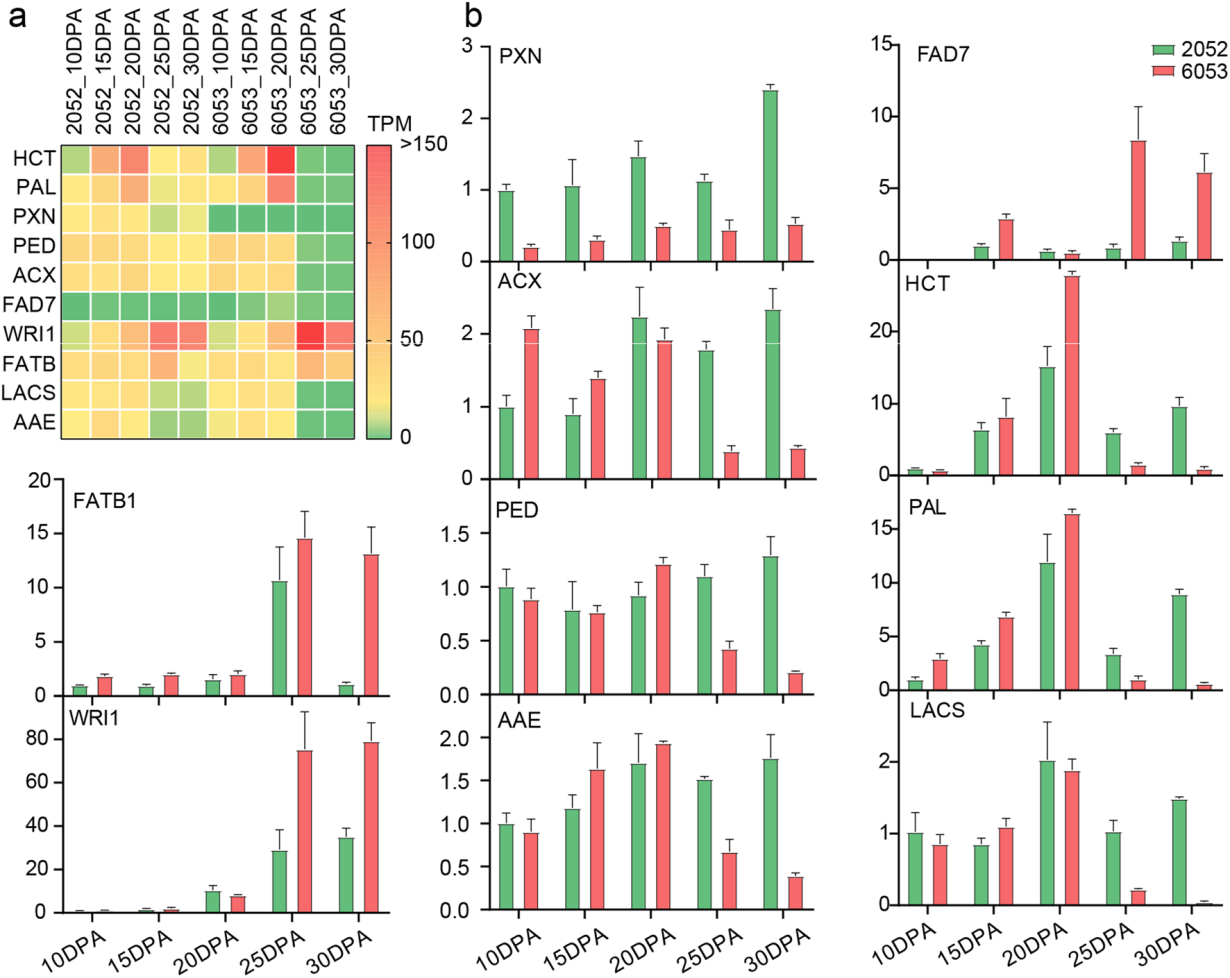
The RNA-seq expression level (**a**) and qRT-PCR validation (**b**) of ten candidate lipid-related genes in H6053 and L2052

To verify the accuracy of the RNA-seq data, ten cottonseed oil candidate genes were selected for qRT-PCR analysis. The ten genes were associated with flavor and oil biosynthesis, such as genes *PAL* and *HCT* involved in phenylpropanoid and flavonoid metabolism, as well as genes *FAD* and *FATB1* involved in oil biosynthesis. The qRT-PCR results showed that the relative expression of the ten genes in H6053 and L2052 (Fig. 6a) were almost consistent with the qRT-PCR expression level (Fig. 6b). In other words, the RNA-seq data were reliable and conducive for the further analysis in this study.

## Discussion

Cottonseed, as a prominent by-product of cotton, plays an important role in oil yield, yet there are few studies focused on cottonseed oil content to date. Comparative transcription analyses of high and low oil cotton materials offers an effective approach to investigate differentially expressed genes and potential candidate genes associated with oil biosynthesis. In the present study, we performed a comprehensive comparative transcriptome analysis of L2052 and H6053. Therefore, a high-resolution gene expression network was generated and provided valuable insights into the genetic basis underlying FA biosynthesis during cottonseed development.

### Candidate genes newly discovered for cottonseed oil biosynthesis

Complex gene regulation network, including by TFs and miRNAs, is responsible for the variance in cottonseed oil content [5, 34, 35]. Cottonseed oil is an unsaturated vegetable oil dominated by linoleic acid [36]. Polyunsaturated FA are catalyzed by desaturases, such as FAD2 introducing a second double bond into oleic acid to form linoleic acid and FAD3 introducing a third double bond into linoleic acid to form α-linolenic acid. In this study, we found that more genes were down-regulated in H6053 than that in L2052 during cottonseed development, and the number of down-regulated genes were significantly increased in '20 DPA vs 25 DPA'. GO and KEGG enrichment analysis confirmed that most of the down-regulated DEGs were involved in phenylpropane and flavonoid biosynthesis pathway during the critical period of cottonseed oil accumulation, and the up-regulated genes were mainly involved in the FA biosynthesis (Fig. 1). Based on these results, some key genes required for the high oil content of *G. hirsutum* were identified, such as TFs (TTG1, TT8, WRI1, and NF-YB6), FA synthesis (FAD2 and FAD3), oil body proteins (OLE1, OLE2, and OLE4), and TAG assembly (GPAT, LPAAT, LPACT, and PDAT). The expression level of the FAD2 gene was found to be consistently high in both the H6053 and L2052, with no significant difference. On the other hand, the expression of the FAD3 gene was notably higher in L2052 compared to H6053. These findings not only support the crucial role of linoleic acid synthesis in seed oil accumulation but also reveal an interesting pattern in gene expression. Specifically, the expression levels of genes associated with oil synthesis were consistently higher during the cottonseed developmental stages (20 DPA to 30 DPA). In contrast, the expression of genes involved in phenylpropanoid and flavonoid synthesis showed a notable decrease during the same period. This indicates a potential negative correlation between the phenylpropanoid/flavonoid synthesis pathways and cottonseed oil accumulation.

### Flavonoid synthesis is antagonistic to cottonseed oil accumulation

Lignin and flavonoid synthesis pathways are two important branching pathways of phenylpropanoid biosynthesis in plants [37]. Hydroxycinnamoyl CoA:shikimate hydroxycinnamoyl transferase (HCT) is a central enzyme of the so-called “esters” pathway to monolignols [38]. It has also been found that lignin was diverted to the flavonoid synthesis pathway, which can increase the flavonoid content in HCT-silence [39]. Cinnamoyl CoA reductase (CCR) catalyzes the reductive reaction to synthesize p-coumaraldehyde, the first step of lignin synthesis [40]. 4-Coumaroyl CoA is a substrate used for the synthesis of monolignols and flavonoids [31]. In this study, the expression of HCT gene was significantly down-regulated at 25 DPA and 30 DPA in H6053 relative to L2052. Moreover, the expression of genes involved in the de novo phenylpropanoid synthesis were also decreased during cottonseed oil biosynthesis, such as gene encoding 4CL and genes TTG1/TT8, which indicates that the decreased synthetic flux of phenylpropane pathway may have contributed to the high cottonseed oil content [9, 41]. Previous studies have shown that catechins are negatively correlated with FA content [30]. Leucoanthocyanidin reductase (LAR), an important enzyme catalyzing leucoanthocyanidin to catechin [42], showed a slightly higher expression level in L2052. Interestingly, this study found that catechin content was positively correlated with linolenic acid content in cottonseed ovule (Additional file 2: Fig. S2d), while negatively correlated with long chain FA content (Additional file 2: Fig. S2c). The result suggested that there may be a competitive relationship between oil synthesis and flavonoid synthesis for energy substances and substrates. In addition, cottonseed oil with a high linoleic acid content is susceptible to oxidation. Hydrogenation is a common method to solve this problem, but hydrogenation can produce trans fatty acids, which are more harmful to the human body than saturated fatty acids. Therefore, an attempt to reduce the flux of phenylpropanoid synthesis in cottonseed may be an option to improve cottonseed oil quality in the future.

### Introgression gene *GhPXN1* plays vital role in cottonseed oil accumulation

Introgression event plays a significant role in the enhancement of cultivars in *G. hirsutum* and is directly associated with agronomic variations [43]. As *G. barbadense* cultivation migrated northward (from South America to the Caribbean and eventually worldwide), hybridization with *G. hirsutum* is believed to have been crucial in reshaping the adaptation and phenotypes of *G. barbadense* [44]. Recently, numerous interspecific crosses between *G. hirsutum* and *G. barbadense* have been developed to identify quantitative trait loci (QTLs) for agronomic traits [45, 46]. For example, stable QTLs *qFL-A03-1*, *qFL-D07-1*, and *qFL-D13-1* were identified by constructing recombinant inbred lines with *G. barbadense* introgressions [47]. Locus *Gb_INT13* in *G. barbadense* may have been derived from *G. hirsutum* [48]. Consequently, investigating the introgression events that occurred between different cotton species is of utmost importance. In this study, a comprehensive investigation was conducted on the introgression events focusing on the *GhPXN1* genes of 218 *G. arboreum* accessions and 419 *G. hirsutum* accessions, including *G. hirsutum* 2052. Result of phylogenetic tree analysis revealed a potential introgression of the *GhPXN1* gene from *G. arboreum* into *G. hirsutum* 2052 (Fig. 5b). The *GhPXN1* gene, encoding a peroxisomal membrane protein, is of significant importance in cottonseed oil content. Previous studies have reported that the overexpression of specific genes, such as *WRI1*, *LEC1*, *LEC2*, and *DGAT1*, can significantly enhance cottonseed oil content [49–51]. Notably, overexpression of *GhPXN1* has been observed to significantly increase the oil content in tobacco leaves in this study. Therefore, *GhPXN1* emerges as a promising key gene associated with cottonseed oil content, holding potential for future research and improvement efforts.

## Conclusion

In this study, our goal was to identify key cottonseed oil candidate causal genes through comparative transcriptome and WGCNA analysis between H6053 and L2052. As a result, several key regulators (enzymes and TFs) required for high oil accumulation in *G. hirsutum* were identified, including TFs (TTG1, TT8, WRI1, and NF-YB6), oil body proteins (OLE1, OLE2, and OLE4), and TAG assembly enzymes (GPAT, LPAAT, LPACT, and PDAT). Moreover, ten key candidate genes were validated by qRT-PCR, and it was found that the oil gene *GhPXN1* was introgressed from *G. arboretum* to *G. hirsutum*. Overexpression of the *GhPXN1* in tobacco significantly reduces oil content compared to empty-vector transformants. This study may facilitate development of cottonseed oils as a biodiesel feedstock, and provide new insight into the regulatory mechanism of high oil production for further metabolic engineering of oil accumulation in cottonseed and other oil plants.

## Materials and methods

### Plant materials and phenotype

Cotton varieties L2052 and H6053, exhibiting significant differences in seed oil content, were carefully selected from a recombinant inbred line (RIL) population derived from *G. hirsutum* AC11 and JK178 in this study. These two cotton genotypes were cultivated in a single-row plot with 18–23 plants, plot length of 4 m, and row spacing of 0.4 m at the Institute of Cotton Research (ICR) of the Chinese Academy of Agricultural Sciences (CAAS) in Anyang, Henan, China. The local recommended guidelines for cotton production were followed for crop management practices. The cottonseed oil content was determined according to the method described by Ma et al. [51]. The BLUP values for cottonseed oil content were estimated using the R package lme4 (https://github.com/lme4/lme4). Data on flavonoid content were extracted from a previous study [30].

### RNA extraction and library construction

Fresh ovules were sampled at various stages of cottonseed development, including 10, 15, 20, 25, and 30 DPA. Each stage consisted of three biological replications, with 5–10 plants per replication. The collected samples were immediately submerged in liquid nitrogen and stored at − 80 °C. Total RNA samples were extracted using TIANGEN column plant RNA extraction kit (TIANGEN, Beijing) and purified with the RNeasy mini kit (QIAGEN, Germantown, MD, USA) according to the manufacturer’s instructions. The quality and quantity of the purified RNA were evaluated using a Nanodrop ND1000 spectrophotometer (Nanodrop Technologies, Wilmington, DE, USA), Qubit 2.0 fluorometer (Life Technologies, Carlsbad, CA, USA), and an Agilent 2100 Bioanalyzer (Santa Clara, CA, USA). Only high-quality RNA samples were used for cDNA library construction, which was performed using the Illumina TruSeq Stranded RNA Kit (Illumina, San Diego, CA, USA) according to the manufacturer's recommended protocols.

### Transcriptome sequencing and DEGs identification

Paired-end sequencing of the purified cDNA fragments was performed on the Illumina HiSeq X platform. The quality of the raw reads was assessed using FastQC (https://github.com/s-andrews/FastQC). Subsequently, the high-quality reads were aligned to the *G. hirsutum* reference genome TM-1 [52] using hisat2 (V2.1.0) [53] with default parameters. The expression levels of genes were quantitatively estimated using the Transcripts Per Kilobase of exon model per Million mapped reads (TPM) method. DEGs were identified using the R package DESeq2 with a cut-off criteria of |log_2_FC |≥ 2 and P-adj ≤ 0.01.

### Construction of gene co-expression network

Gene co-expression network was constructed using the R package WGCNA [54]. Each biological replication was considered as a distinct dataset, resulting in a total of 30 datasets (2 genotypes with 5 stages and 3 replicates). Genes with a sum expression of less than 30 across all samples were excluded. The TOMType parameter was set to unsigned, minModuleSize was set to 200, and mergeCutHeight was set to 0.15. The eigengene value was calculated for each module and used to assess the association with different tissue types. Functional annotation of the genes was performed using GO and KEGG enrichment analysis (http://grand.cricaas.com.cn) [55].

### Expression of oil candidate genes in *Nicotiana benthamiana* fatty acid system

To construct an overexpression system for the *GhPXN1* gene, the full-length coding sequence (CDS) was amplified. The amplified CDS was then inserted into the 2300 vector (Invitrogen) to establish the overexpression system. The Agrobacterium strain of Saccharomyces cerevisiae (Weidi Biotechnology Co., Shanghai, China) was employed. As a control, the empty-vector 2300-GFP was also constructed. In order to induce transient expression in tobacco, the same method described by Ma et al. [56] was followed for both the *GhPXN1* overexpression system and the control (CK).

### qRT-PCR

To verify the accuracy of RNA-seq data, a subset of ten DEGs was chosen for qRT-PCR validation. Total RNA samples used for RNA-seq were employed. cDNA synthesis was performed using the HiScript III Q RT SuperMix for qPCR reverse transcription kit (Vazyme Biotech Co., Ltd.). Subsequently, qRT-PCR verification was carried out using the ChamQ Universal SYBR qPCR Master Mix Kit (Vazyme) following the established protocol [57]. The primer sequences for the real-time PCR are provided in Additional file 1: Table S5. The qRT-PCR reaction started with initial denaturation at 95 °C for 30 s, followed by 40 cycles of denaturation at 95 °C for 10 s, annealing and extension at 60 °C for 30 s, and a final extension at 12 °C for one minute. The reference gene *HISTONE3* (AF024716) was used for normalization. The experimental design incorporated three biological replicates for each gene, and the relative expression levels were calculated using the 2^−ΔΔCT^ method [58].

### Supplementary Information

**Additional file 1: Table S1.** The contents (%) of different fatty acid components in high (6053) and low (2052) oil cottonseed. **Table S2.** The KEGG enrichment analysis of down-regulated genes between '20 vs 25 DPA' in 6053 and 2052. **Table S3.** Genes involved in oil biosynthesis in development cottonseed of 6053 and 2052. **Table S4.** Expression profile of oil related TFs in cottonseed development of *G. hirsutum *6053 (high-oil) and 2052 (low-oil). **Table S5.** The designed primers of genes encoding the key enzymes involved in lipid biosynthesis for qRT-PCR.

**Additional file 2: Figure S1.** **a** Principal components analysis (PCA) of RNA-seq data. **b** Multiple comparisons between the genotypes 2052 and 6053 at various stages of ovule development. The numbers around the arrows indicate the number of differentially expressed genes for the specified comparisons. Red, up-regulation; blue, down-regulation. c The distribution and KEGG functional enrichment analysis of up-regulated (**c**) and down-regulated (**d**) genes. **Figure S2.** **a** The distribution of catechin in natural populations. **b** Correlation analysis of catechin and linoleic acid. **c** Correlation analysis of catechin and oil content. d Correlation analysis of catechin and percentage content of fatty acids. **Figure S3.** **a** The expression profile of oil-related transcription factors in development cottonseed of genotype 6053 and 2052. **b** The expression profile of NF-YB6 and WRI1. peaked at 25DPA. **Figure S4.** KEGG function enrichment analysis of four WGCNA modules (MEbrown, MEdreen, MEmagenta, and MEpink).

## Data Availability

Raw data of the transcriptome analyzed in this work are available at NCBI Sequence Read Archive (https://www.ncbi.nlm.nih.gov/sra) with the accession number PRJNA803743.
