# Supplementary material for: Oil candidate genes in seeds of cotton (Gossypium hirsutum L.) and functional validation of GhPXN1
Source: Biotechnol Biofuels Bioprod. 2023 Nov 6;16:169. doi: 10.1186/s13068-023-02420-1 (PMC10629180; doi:10.1186/s13068-023-02420-1)
Supplement: Supplementary file 2 — Additional file 2: Figure S1. a Principal components analysis (PCA) of RNA-seq data. b Multiple comparisons between the genotypes 2052 and 6053 at various stages of ovule development. The numbers around the arrows indicate the number of differentially expressed genes for the specified comparisons. Red, up-regulation; blue, down-regulation. c The distribution and KEGG functional enrichment analysis of up-regulated (c) and down-regulated (d) genes. Figure S2. a The distribution of catechin in natural populations. b Correlation analysis of catechin and linoleic acid. c Correlation analysis of catechin and oil content. d Correlation analysis of catechin and percentage content of fatty acids. Figure S3. a The expression profile of oil-related transcription factors in development cottonseed of genotype 6053 and 2052. b The expression profile of NF-YB6 and WRI1. peaked at 25DPA. Figure S4. KEGG function enrichment analysis of four WGCNA modules (MEbrown, MEdreen, MEmagenta, and MEpink). [file 13068_2023_2420_MOESM2_ESM.docx]

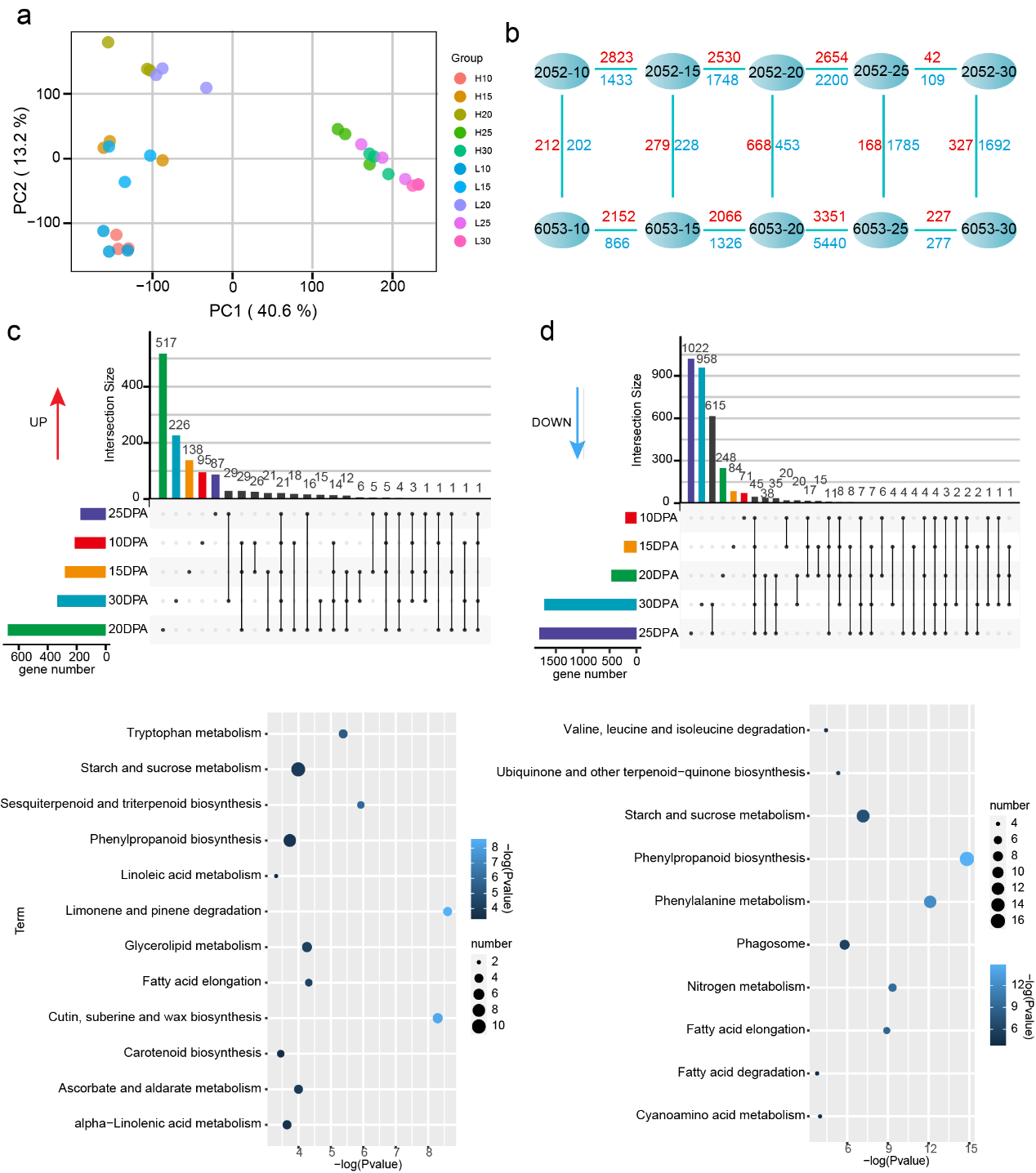


Figure S1 **a.** Principal components analysis (PCA) of RNA-seq data. **b** Multiple comparisons between the genotypes 2052 and 6053 at various stages of ovule development. The numbers around the arrows indicate the number of differentially expressed genes for the specified comparisons. Red, up-regulation; blue, down-regulation. **c** The distribution and KEGG functional enrichment analysis of up-regulated (**c**) and down-regulated (**d**) genes.


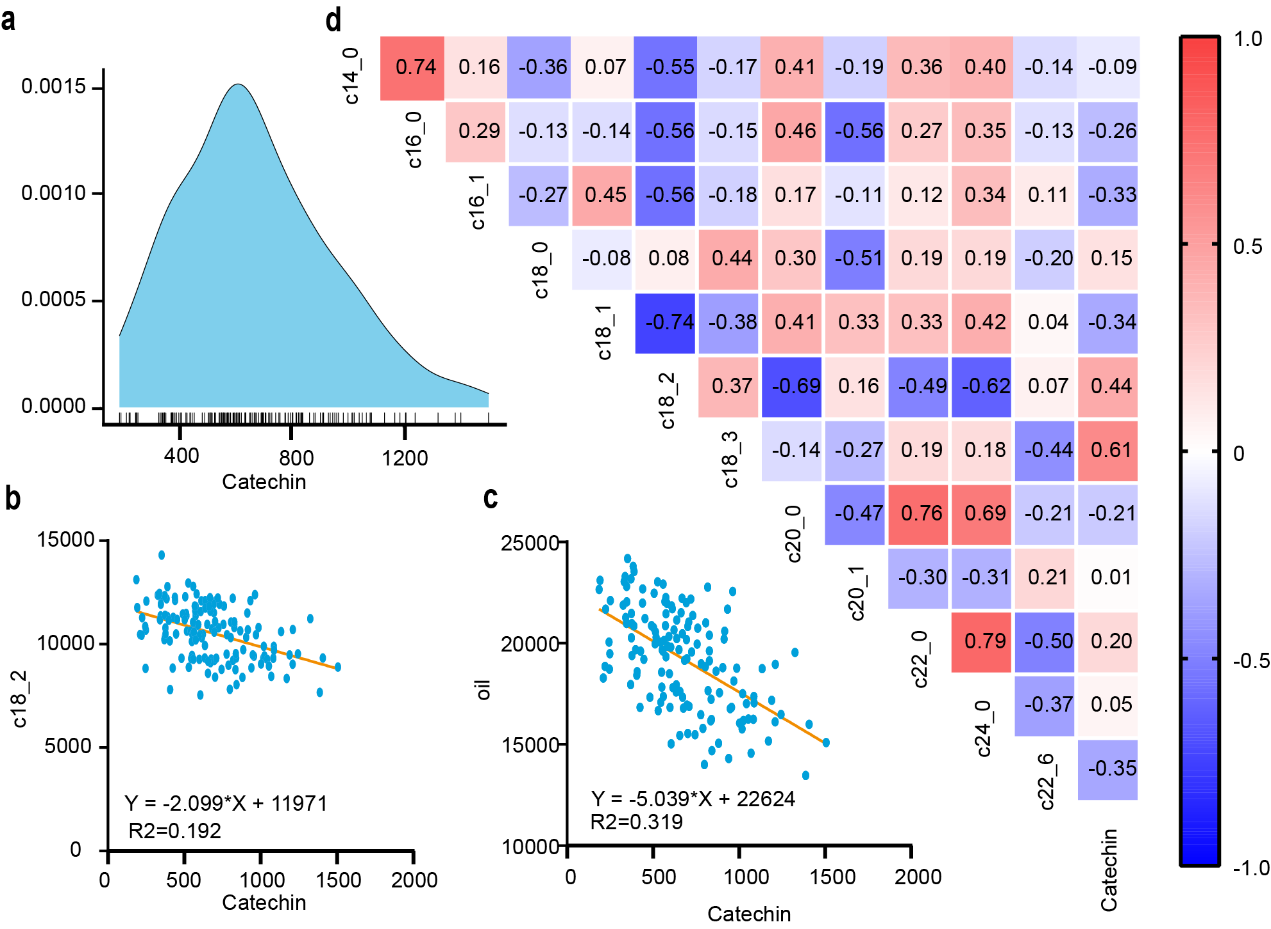


Figure S2 **a** The distribution of catechin in natural populations. **b** Correlation analysis of catechin and linoleic acid. **c** Correlation analysis of catechin and oil content. **d** Correlation analysis of catechin and percentage content of fatty acids.


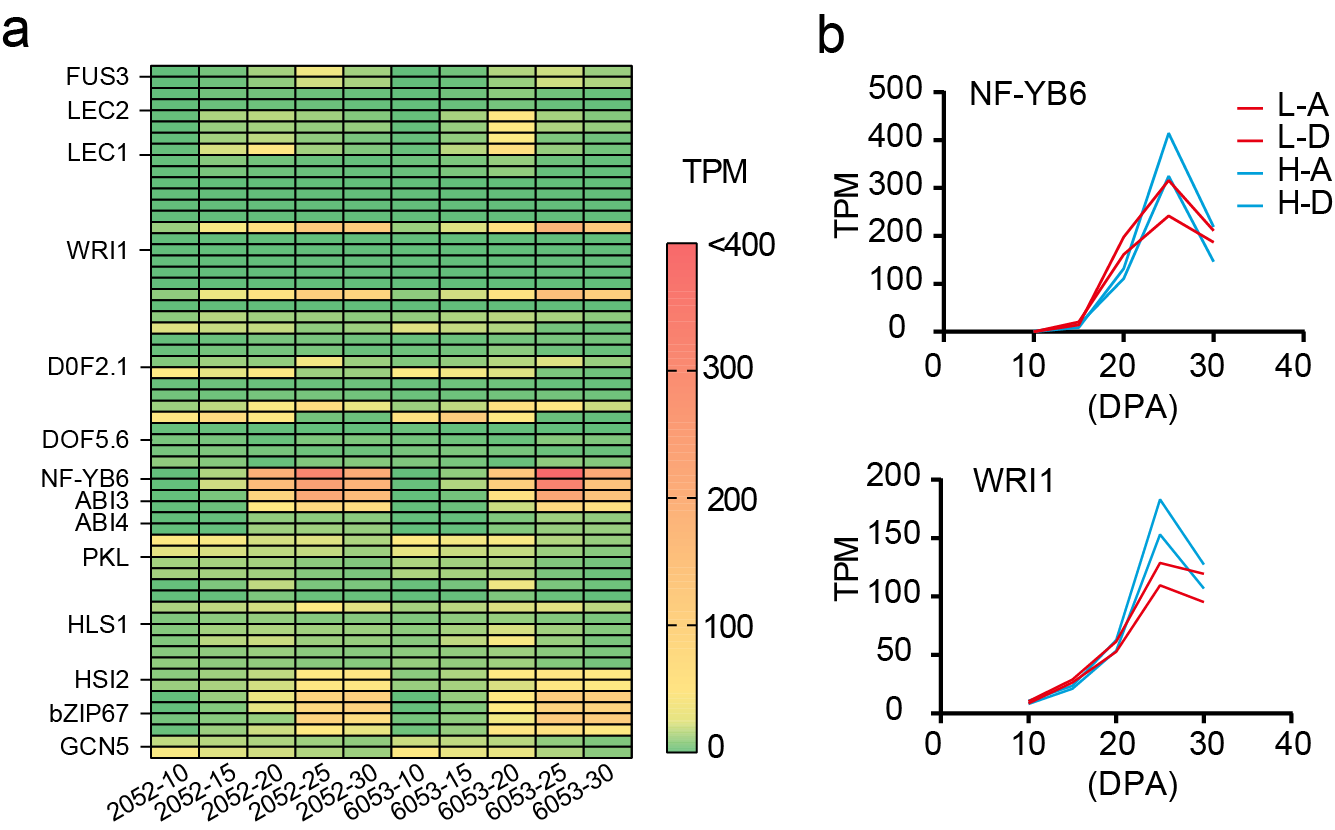


Figure S3 **a** The expression profile of oil-related transcription factors in development cottonseed of genotype 6053 and 2052. **b** The expression profile of NF-YB6 and WRI1. peaked at 25DPA.


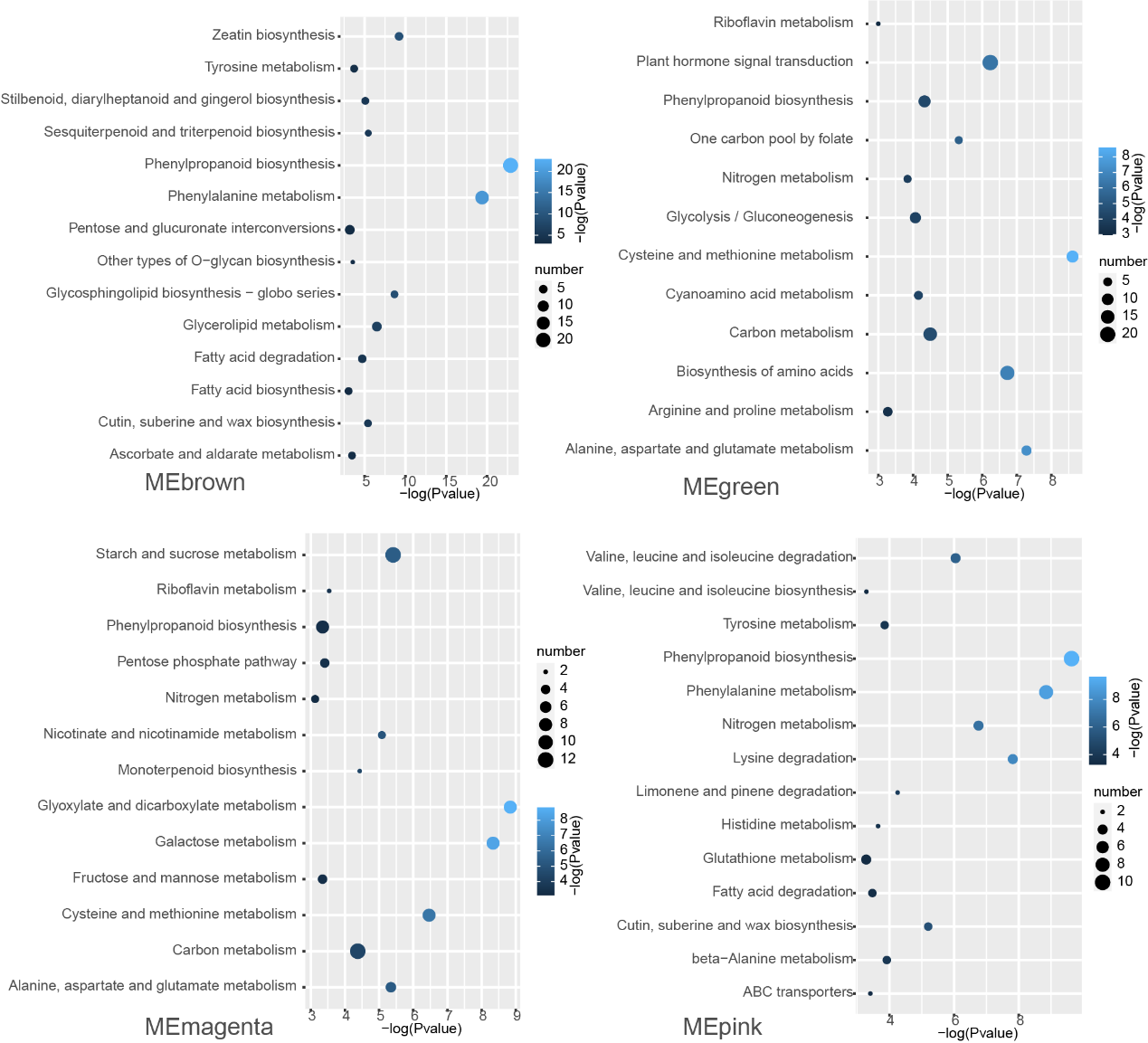


Figure S4 KEGG function enrichment analysis of four WGCNA modules (MEbrown, MEdreen, MEmagenta, and MEpink).
